# Effectiveness of a group diabetes education programme in underserved communities in South Africa: pragmatic cluster randomized control trial

**DOI:** 10.1186/1471-2296-13-126

**Published:** 2012-12-24

**Authors:** Bob Mash, Naomi Levitt, Krisela Steyn, Merrick Zwarenstein, Stephen Rollnick

**Affiliations:** 1 Family Medicine and Primary Care, Stellenbosch University, Box 19063, Tygerberg, 7505, South Africa; 2 Diabetic Medicine and Endocrinology, University of Cape Town and Chronic Diseases Initiative in Africa, Faculty of Health Sciences, University of Cape Town, P.Bag X3, Observatory 7935, Room J47 – 85, Old Groote Schuur Hospital Building, Cape Town, South Africa; 3 Chronic Diseases Initiative in Africa, Faculty of Health Sciences, University of Cape Town, P.Bag X3, Observatory, Room J47 – 85, Old Groote Schuur Hospital Building, Cape Town, 7935, South Africa; 4 Institute for Clinical Evaluative Sciences and Department of Health Policy, Management and Evaluation, University of Toronto, G1 06, 2075 Bayview Avenue, Toronto, ON, M4N 3M5, Canada; 5 Professor of Health Care Communication, Department of Primary Care and Public Health, Cardiff University, Neuadd Meirionnydd Heath Park, Cardiff, WALES, CF14 4YS, UK

**Keywords:** Diabetes, Group education, Health education, Motivational interviewing, Mid-level health workers, South Africa, Primary care

## Abstract

**Background:**

Diabetes is an important contributor to the burden of disease in South Africa and prevalence rates as high as 33% have been recorded in Cape Town. Previous studies show that quality of care and health outcomes are poor. The development of an effective education programme should impact on self-care, lifestyle change and adherence to medication; and lead to better control of diabetes, fewer complications and better quality of life.

**Methods:**

**Trial design:** Pragmatic cluster randomized controlled trial

**Participants:** Type 2 diabetic patients attending 45 public sector community health centres in Cape Town

**Interventions:** The intervention group will receive 4 sessions of group diabetes education delivered by a health promotion officer in a guiding style. The control group will receive usual care which consists of ad hoc advice during consultations and occasional educational talks in the waiting room.

**Objective:** To evaluate the effectiveness of the group diabetes education programme

**Outcomes:** Primary outcomes: diabetes self-care activities, 5% weight loss, 1% reduction in HbA1c. Secondary outcomes: self-efficacy, locus of control, mean blood pressure, mean weight loss, mean waist circumference, mean HbA1c, mean total cholesterol, quality of life

**Randomisation:** Computer generated random numbers

**Blinding:** Patients, health promoters and research assistants could not be blinded to the health centre’s allocation

**Numbers randomized:** Seventeen health centres (34 in total) will be randomly assigned to either control or intervention groups. A sample size of 1360 patients in 34 clusters of 40 patients will give a power of 80% to detect the primary outcomes with 5% precision. Altogether 720 patients were recruited in the intervention arm and 850 in the control arm giving a total of 1570.

**Discussion:**

The study will inform policy makers and managers of the district health system, particularly in low to middle income countries, if this programme can be implemented more widely.

**Trial register:**

Pan African Clinical Trial Registry PACTR201205000380384

## Background

Diabetes is a significant contributor to the burden of disease in South Africa [1] and the prevalence in Africa is expected to increase by 80% over the next 15 years [2]. Self-reported prevalence rates for diabetes of 2.4% in men and 3.7% in women have been reported in South Africa [3]. However, studies in the Western Cape, one of the nine provinces of South Africa, suggest rates in urban areas as high as 33% [4].

Approximately 80% of the 3,1 million population in the Cape Town Metropole are uninsured and rely on public sector facilities to manage their diabetes. Several previous studies in the Western Cape have illustrated the poor quality of care and outcomes for diabetic patients [5,6]. Almost 80% of patients were uncontrolled (HbA1c≥7%) in an audit of type 2 diabetes in Cape Town’s public sector in 2011 [6]. Deficiencies in knowledge and self-care amongst patients and the inability of primary care providers to ameliorate this, have been identified as part of the problem. The population served by the public sector is characterized by low socio-economic status, low levels of education and low health literacy. The population served come from historically disadvantaged communities and speak mainly Afrikaans or Xhosa.

Primary care services in the country are largely nurse-led with the support of doctors. Other health workers, appropriate to the management of diabetes, such as dieticians and podiatrists, are usually not available. A variety of mid-level workers, such as health promoters, have been trained and employed in community health centres. Currently the education of diabetic patients and support of self-care has been left to the varied initiatives of individual health workers and there is no structured programme of education for people with diabetes in the Western Cape. Chronic care teams have identified that the health promoter should be the key person in delivering such a programme [7].

Community health centres in the Western Cape are usually found in larger metropolitan areas or rural towns. Diabetic patients are often seen in such large numbers that a specific day is set aside each week for them to attend a diabetic “club”. At a given health centre patients are usually scheduled to attend the “club” as a group and are given appointments to be seen for review every 3 months.

During 2006–2008 the investigators were involved in an appreciative inquiry project to improve the annual review of the diabetic patients in the Cape Town Metropole. During this project the quality of care in the annual audit in terms of assessment of HbA1c, serum creatinine and cholesterol, retinal screening, foot screening and calculation of body mass index significantly improved [7]. At the end of this project staff articulated the need for a better approach to diabetes education and collaborated in designing the content of a more structured programme to be delievered by health promoters [7].

Health promoters have a secondary school education up to at least Grade 8 and once employed have additional training in the knowledge and skills required to deliver health education messages and promote health. There are currently 120 health promoters in the Province and the policy of the Department of Health is to have a health promoter at every community health centre. A recent study showed that the current health promoters have a good knowledge of key diabetes education messages for patients [8].

Although a variety of individual and group educational materials are available from non-government organizations and pharmaceutical companies, no materials are officially disseminated or recommended by the national or provincial Department of Health.

The relationship between health care provider and patient is recognized to have an important influence on patient understanding and adherence [9]. Motivational interviewing has been recommended as a more skilful guiding approach to eliciting lifestyle change and promoting self-care [10] and a recent systematic review concluded that it out-performs traditional advice-giving in 80% of studies [11,12]. Professors Rollnick and Mash are members of the International Network of Motivational Interviewing Trainers and have experience with training and researching in this area [10,13]. Studies of individually-delivered motivational interviewing for diabetic patients have produced promising results [14,15]. A recent study of individual motivational interviewing for the prevention of diabetes also demonstrated a significant effect on achieving 5% weight loss [16].

Group interactions have been found to be effective in diabetes education [17] and local chronic care staff have indicated that this is the most practical approach in their very busy health centres [7]. A systematic review of group education in diabetes concluded that “The results of the meta-analyses in favour of group-based diabetes education programmes were: reduced glycated haemoglobin at four to six months (1.4%; 95% confidence interval (CI) 0.8 to 1.9; P < 0.00001), at 12–14 months (0.8%; 95% CI 0.7 to 1.0; P < 0.00001) and two years (1.0%; 95% CI 0.5 to 1.4; P< 0.00001); reduced fasting blood glucose levels at 12 months (1.2 mmol/L; 95% CI 0.7 to 1.6; P < 0.00001); reduced body weight at 12–14 months (1.6 Kg; 95% CI 0.3 to 3.0; P = 0.02); improved diabetes knowledge at 12–14 months (SMD 1.0; 95% CI 0.7 to1.2; P < 0.00001) and reduced systolic blood pressure at four to six months (5 mmHg: 95% CI 1 to 10; P = 0.01). There was also a reduced need for diabetes medication (odds ratio 11.8, 95% CI 5.2 to 26.9; P < 0.00001; RD = 0.2; NNT = 5). Therefore, for every five patients attending a group-based education programme we could expect one patient to reduce diabetes medication” [17]. Motivational interviewing in group format is a relatively new development; 12 published reports have emerged [18,19], which include three randomized trials and one study in the diabetes field [20].

Despite evidence of the effectiveness of group diabetes education all of the trials have been conducted in high-income countries and by health professionals such as doctors or nurses. This trial will be the first in an African context and delivered by mid-level health workers. In addition the incorporation of group motivational interviewing will add to a small evidence base on this topic.

If the study demonstrates effectiveness of this educational intervention then it can be implemented throughout the Western Cape and may well be replicated in the rest of the country and possibly within the southern African Region. The study intends to inform policy makers and managers of the district health services and help them decide whether to implement the programme more widely.

### Aim and objectives

Aim: To evaluate, by means of a pragmatic cluster randomized controlled trial, the effectiveness of a group diabetes education programme delivered by trained health promoters with a guiding (motivational interviewing) style, in community health centres in Cape Town, South Africa.

Objective 1: To evaluate effectiveness by assessing group changes in the following:

*Primary outcomes:* improved diabetes self-care activities, 5% weight loss, HbA1c reduction of 1 percentage point

*Secondary outcomes:* improved diabetes-specific self-efficacy, locus of control, mean blood pressure, mean weight loss, mean waist circumference, mean HbA1c, mean total cholesterol and quality of life

Objective 2: To evaluate fidelity to the educational programme and motivational interviewing by use of audiotapes and scoring health promoters with the Motivational Interviewing Integrity Code.

Objective 3: To explore the experiences of the health promoters with regards to their training and delivery of the diabetes education programme.

Objective 4: To explore the experiences of patients who attend the diabetes education sessions.

## Methods

### Study design

Pragmatic cluster randomized controlled trial with additional qualitative and quantitative process evaluations. The CONSORT statement on pragmatic trials was used to assist with the design [21].

### Target population

Patients with Type 2 Diabetes attending 45 Community Health Centres in the Cape Town Metropole were the target population. Thirty eight health promoters are currently employed within these facilities.

### Sample size

Data from a previous study in the same population (n=450, 18 clinics) showed that the mean HbA1c was 8.8% (SD 3.3) and intraclass correlation 0.1 [22]. Similarly the mean weight was 78.2Kg (SD 16.7) and intraclass correlation 0.05. These figures were used to calculate the sample and cluster size for a 5% weight reduction and a 1% reduction in HbA1c. Based on a level of significance of 0.05 and a power of 0.8 the study required 17 clusters in each arm with 40 patients per cluster. The total sample size therefore would be 34 clusters (health centres) and 1360 patients.

### Sample selection process

Community health centres that agreed to participate were randomly allocated by computer generated random numbers to either control or intervention groups. All type 2 diabetic patients attending the selected health centre on the recruitment days were invited to participate in the study. Recruitment days were when the health centre had a diabetic club. Centres were visited weekly until the sample of 40 patients per health centre was obtained. Altogether 720 patients were recruited in the intervention arm and 850 in the control arm giving a total of 1570.

### Inclusion and exclusion criteria

Inclusion: All type 2 diabetic patients who gave consent, regardless of the type of medication (oral and/or insulin) or time since diagnosis.

Exclusion: Type 1 diabetic patients, those who refused consent, those who were judged by the clinical nurse practitioner or medical officer as unable to participate in the intervention (for example due to acute illness, mental illness, dementia or another justifiable reason).

### Design of intervention

The intervention was developed by the researchers with assistance from a diabetes nurse educator and social scientist with an interest in behaviour change counseling. The sessions were piloted with a group of diabetic patients attending Groote Schuur Tertiary Hospital.

The following overall structure was suggested by the chronic care teams (including health promoters) in a previous study [7]. Patients should receive 4 educational sessions each lasting between 20–60 minutes. Sessions should be offered when the patients are scheduled for a routine visit to the health centre by a health promoter. Groups should have between 10 and 15 people who would remain together throughout the programme:

Session 1: Understanding diabetes

Session 2: Living a healthy lifestyle

Session 3: Understanding the medication

Session 4: Preventing complications

The researchers reviewed a number of materials for group education and found the Conversation Map™ the most congruent with the design of the intervention [23-25]. The Conversation Map™ materials were piloted in a rural town to see which aspects were appropriate for the local context [26]. A number of the group activities, such as working with myths and facts cards, recommended in the Conversation Map™ material were adapted for the local context. The researchers also developed new graphic materials to help patients understand the patho-physiology of diabetes as well as the effect of medication and self-care activities. Pictures were developed to illustrate portion size and food choices. All these pictures were then designed and printed in the format of a flip chart. A comprehensive set of food cards illustrating local South African foods and which could be used in group activities were also purchased. Patient education materials on foot care, coping with stress, alcohol and smoking cessation were also developed or sourced locally.

The sessions were designed to be congruent with a guiding communication style [10]. This style was intended to include the following characteristics:

Collaboration: Both health promoters and patients should contribute substantially to the group discussion

Empathy: Health promoters should demonstrate active listening skills and their understanding of the patient’s perspective, particularly through the use of summaries.

Support for autonomy: Health promoters should promote a sense of choice and control over behaviour change

Evocation: Health promoters should elicit change talk and possible solutions from the group members

Direction: Health promoters should manage time and keep aligned with the intended content and purpose of the sessions

It was also recognized that diabetes education often involves a significant component of information and therefore strategies to exchange information rather than just transfer it were taught. In particular the use of elicit-provide-elicit was emphasized as a strategy and in fact the sessions themselves were structured according to this model. This model involves the following three steps in a cyclical process [10].

Elicit either the groups prior knowledge or what they are most interested in learning about with regard to a specific topic

Provide the group with information in a neutral way that builds on what they already know or addresses what they are most interested in

Elicit how group members will make sense of or apply this information personally

The researchers recognized that health promoters were used to delivering health education in a directing style, often in quite difficult circumstances. This directing style was characterized by an authoritarian, expert role that told patients what they should be doing. Educational talks were often given to the whole reception area where health promoters had to shout over staff and patients waiting to be seen. The educational model developed in this study was therefore quite a shift from what health promoters were used to. The goals therefore in terms of communication skills were kept as simple as possible. The training manual with more detailed information on the sessions is available as a Additional file 1.

### Control group

The control patients received usual education at the health centre. Usual education consisted of ad hoc educational talks in the reception area or club room as well as any individual counseling that providers might have time for in the consultation.

### Training of health promoters

Health promoters were trained in an initial 4 day workshop which focused on the overall structure of the sessions, communication style and skills, diabetes knowledge and the first two sessions. Training was conducted in a similar small group educational process with the trainers modeling the same skills expected of the health promoters when they educated patients. Following the initial workshop health promoters began the education immediately and a further 2-day workshop was held 2 months later to reinforce the initial training and introduce the last 2 sessions. The researcher who evaluated their fidelity to the intervention visited each health centre at least twice and gave some feedback to the health promoters after the sessions.

### Implementation of intervention

At the end of the HPO’s initial training a number of logistical issues were addressed. These included identifying the room where sessions could take place or if no space was available in the health centre a suitable local venue such as a library or community hall. Of the 17 randomly selected health centres only 13 had health promoters currently employed and therefore 4 of the health promoters agreed to offer the intervention at two sites.

Following recruitment the patients at each health centre were grouped to time the educational sessions on the same date as their routine attendance for medication. Patients were sent bulk SMS reminders of the date and time of their educational sessions and health promoters were encouraged to call the patients prior to the meetings to remind and motivate them. A once off shopping voucher for a local supermarket was offered as an incentive to attend the sessions (this was equivalent to $2). Letters were sent to all those without a phone and to the pharmacist asking for medication to be handed out at or after the educational session. Attendance certificates were available for those who were working. Health promoters were also provided with glucometers so they could test patient’s glucose at the sessions in the hope that this would also encourage attendance.

### Data collection process

Data was collected at baseline and 12 months later. Data collection teams were employed to visit the health centres over a period of 4 weeks and consisted of a nurse and field workers. Nurses were employed to collect blood and take physical measurements and all members completed the questionnaires with patients. Standard operating procedures were used in measuring weight (electronic scales), waist circumference (tape measure) and blood pressure (Omron digital blood pressure monitor). HbA1c and total cholesterol were measured by one laboratory under the National Health Laboratory Service where quality control measures were in place. The data collection teams received a 1-day training workshop prior to the data collection periods and were supervised daily by the project co-ordinator.

It was not possible to blind the health promoters, patients or data collection teams as to whether the health centre was a control or intervention site.

### Data collection tools

The following data was collected from participants and their medical records at baseline: Age, sex, duration of diabetes, medication used and medical history for concomitant diagnoses and complications. Medication use and new diagnoses were also recorded at follow up.

Self-care activities were measured using a questionnaire that separately scores diet, exercise, foot care, smoking and medication use. This validated questionnaire had previously been used successfully in the South African context [27].

Locus of control measures the patient’s belief in their ability to control their illness (internal locus of control) as opposed to a belief that their illness is outside their own control and primarily in the hands of others (external locus of control) or that control is a matter of luck (chance locus of control). Group education using a patient-centred approach has been shown to increase ones internal locus of control, which itself is linked to the likelihood of behaviour change. A specific questionnaire that measures diabetic locus of control has been developed and was used in this study [28].

Self-efficacy is a measure of the patient’s actual confidence in their ability to perform self-care activities. A simple measure of diabetic self-efficacy has been developed by Stanford University’s study on Diabetes Self-Management [29]. Enhancing self-efficacy is one of the key principles of motivational interviewing and is linked to the likelihood of actual behaviour change [10]. The Stanford questionnaire was contextualized and used to measure self-efficacy.

Diabetes quality of care was measured using a questionnaire that has previously been used in the South African context for Type 2 Diabetic patients [30]. Quality of life is an important health outcome that may be impacted by psychosocial factors, complications, duration of diabetes, demographic variables, gender, type of diabetes, glycaemic control and treatment regimes [31].

### Process evaluation

Fidelity to the planned educational programme and to the communication style was assessed by observing 36 randomly selected group sessions. Sessions were stratified to ensure that each site and session was sampled equally. The observer noted the extent to which the session followed the intended content and process and also made additional field notes. Sessions were recorded on audiotape and subsequently evaluated using the Motivational Interviewing Treatment Integrity Coding, which is a validated tool for assessing proficiency in MI [32]. This tool determines whether the counselor achieved beginning proficiency in MI.

The health promoters experience was evaluated by means of three focus group interviews that were facilitated by an independent researcher. The initial focus group was held immediately after the training, the second was held mid-intervention and the third after all the education had been completed. The patient’s experience was evaluated by means of in depth interviews with one patient from each of the health centres in the intervention group who had attended at least 3 of the sessions. Interviews were also conducted by an independent researcher in the patient’s home after the educational sessions were completed. The qualitative data from these interviews was transcribed verbatim and analysed using the framework method and Atlas-ti software.

### Data analysis

Intention-to-treat analysis will evaluate the primary and secondary outcomes. Any missing baseline data will be imputed using the Markov chain Monte Carlo approach. Missing status at follow-up will be modelled on baseline covariates and randomised group using logistic regression. Inverse probability weighting will be used for the final trial analysis. Models for comparing continuous outcomes will use linear regression and for categorical outcomes will use logistic regression with adjustment for baseline covariates and clustering.

### Timeline

Baseline data collection took place in September-December 2010. The intervention was delivered between October 2010 and April 2011. Follow up data collection took place in September-December 2011. Data capture and cleaning were completed by February 2012 and we are now busy with data analysis.

## Discussion

Although group diabetes education has been shown in systematic reviews to be effective these studies are mostly from resource rich countries with more developed primary care systems [17]. The South African primary care system is struggling to develop in the post-Apartheid era while simultaneously battling with a quadruple burden of disease in the form of HIV/AIDS, injury and violence, high maternal and child mortality and a growing epidemic of non-communicable diseases [1]. The Western Cape probably has the best resourced primary care system in the country, but even there health workers complain of long hours, burnout and depression [33]. Nationally the government is committed to the re-vitalisation of primary care over the next few years [34]. In this context we need to develop approaches to diabetes education that can work in our resource constrained and pressurized environment. This trial intends to evaluate one such approach as suggested by the primary care providers involved in chronic care [7]. The trial is pragmatic in the sense that the intervention is conducted under the organizational and clinical strengths and weaknesses of the current primary care service. It is also innovative in developing a model of group motivational interviewing that is intended to be delivered by a mid level health worker who themselves may only have basic education. Nevertheless in our context task shifting is common and much is expected of such mid level health workers. Group motivational interviewing is a relatively new field and little has yet been published on the topic.

The Department of Health was a partner in the development of the research proposal with the intention that, should the intervention be effective, it can be implemented throughout the Western Cape. The research team maintains links with the Director for Public Health, Deputy Director for Chronic Diseases, Director of District Health Services and the Human Resource and Development Directorate. The intervention being studied is congruent with the newly developed Provincial Policy on Chronic Diseases in the Western Cape Department of Health. The Department of Health is also a partner in the Chronic Disease Initiative in Africa which is a supporting institution. The Western Cape Province has a track record of developing innovations that are later taken up by the National Department of Health. The intervention being tested is also congruent with national policy and we anticipate interest in further implementation if it is effective.

The Chronic Disease Initiative in Africa has a more regional vision and if the intervention is effective will assist with the dissemination of the programme. The International Diabetes Foundation is also the key funder of the study and will disseminate any useful learning to the international community.

## Abbreviations

AIDS: Acquired Immune Deficiency Syndrome; CI: Confidence Interval; HbA1c: Glycosylated Haemoglobin; HIV: Human Immunodeficiency Virus; MI: Motivational Interviewing.

## Competing interests

The authors declare that they have no competing interests.

## Authors’ contributions

All authors contributed to the research proposal described in this article. BM is the principal investigator with particular expertise in primary care and motivational interviewing. DL has particular expertise in clinical diabetes and KS in non-communicable chronic diseases epidemiology. MZ has expertise in pragmatic clustered randomized trial design and contributed to the CONSORT statement in this area. SR is an international expert in motivational interviewing. All authors read and approved the final manuscript.

## Authors’ information

BM co-ordinates a network of motivational interviewing practitioners, trainers and researchers in southern Africa. DL and KS are the directors of the Chronic Diseases Initiative in Africa which also supported this study. MZ is a South African and used to be head of the Health Systems Research Unit at the SA Medical Research Council prior to his current position at the University of Toronto. SR is also a South African and is one of the founding fathers of motivational interviewing as an approach to behavior change counseling.

## Pre-publication history

The pre-publication history for this paper can be accessed here:

http://www.biomedcentral.com/1471-2296/13/126/prepub

## Supplementary Material

Additional file 1 **Group diabetic education.** Training manual for health promoters. Click here for file
