# Supplementary material for: Effectiveness of a group diabetes education programme in underserved communities in South Africa: pragmatic cluster randomized control trial
Source: BMC Fam Pract. 2012 Dec 24;13:126. doi: 10.1186/1471-2296-13-126 (PMC3560091; doi:10.1186/1471-2296-13-126)
Supplement: Additional file 1 — Group diabetic education. Training manual for health promoters. [file 1471-2296-13-126-S1.docx]

**Group diabetic education: Training manual for health promoters**

# Format of intervention

The intervention will consist of 4 educational sessions and each community health centre will have 4 groups of diabetic patients. For a specific group of patients the sessions will take place at the community health centre, once a month for four months (October 2010, November 2010, February 2011, March 2011). They will most likely take place in the afternoon at a time agreed with the health promoters. Two hours should be allocated for each session.

The patients with type 2 diabetes, recruited for the intervention, will include those patients newly diagnosed with diabetes, those patients under long term management at the clinic facility and those patients on insulin.

Diabetic patients usually come to the clinic for a proper assessment every 3-6 months. In the intervening months they come to the pharmacy to collect their medication. It should be possible to arrange the intervention so that it coincides with the date on which they would usually come to collect their medication.

September – Recruitment and club / assessment visit

October – Session 1 and Medication

November – Session 2 and Medication

December or January – Club / assessment visit

February – Session 3 and Medication

March – Session 4 and Medication

You will need to arrange with the pharmacy that their medication be given before or after the session and that the patients do not have to spend additional time waiting in the pharmacy queue. This was discussed with the facility managers by Hilary Rhode.

Patients will receive a certificate from the University for participating in the educational sessions at the end of the 4^th^ session.

# Content and style of intervention

Facilitation of the group process may need a shift in the overall style of communication from what has been termed a “directing” style to a “guiding” style. The guiding style is thought to be more appropriate and effective when helping people to change their behavior.

Education in the health centres has usually followed a directing style that can be characterized as “giving a talk” to patients in a way that information is transferred in only one direction, the speaker is seen as an expert or authority figure, whose advice must be followed by the patients. When this advice is not followed patients are often seen as irresponsible and difficult.

The guiding style, by contrast, can be characterized as “helping people to make difficult choices” through a collaborative interaction that recognizes the expertise of the patient in managing their own life, shares information tailored to the patient’s needs and interests, and respects the decisions that patients make. The guiding style involves all of the following:

*Collaboration* – working together with the patients, rather than telling them what to do in an authoritarian or one-sided expert role. It is like two experts collaborating, the one is an expert in diabetes and the other an expert in their own life circumstances and situation.

*Empathy* – carefully listening to the different viewpoints in the group and making this understanding explicit by the use of summaries

*Evocation* – drawing out solutions and ideas about change from the group often through the use of open questions, rather than telling them what to do or think.

*Support for autonomy* – supporting people in taking control of their diabetes and making their own choices. Respecting people’s choices, even when you would like them to be different.

*Direction –* facilitating the group in such a way that the focus on the various topics is maintained and the group follows a clear process, with a clear purpose.

The 4 sessions will be based on the suggested topics which arose from the Appreciative Inquiry research process which took place with the primary health care teams in 2006-2008:

1. Understanding Diabetes
2. Living a healthy lifestyle
3. Understanding the medication
4. Preventing complications

Each session will start with a brief INTRODUCTION that will engage the group with the topic and each other. In the first session this will focus on introducing people and the topic of diabetes. In subsequent sessions it will focus on people giving brief feedback on the successes they have had with implementing their goals during the previous month and any lessons they learnt about how to look after their diabetes better. This opening discussion will focus on the progress people have made or the positive experiences they have had with behavioural change, rather than the difficulties.

In each session, you will then facilitate a group discussion about the management of diabetes, based on an essential construct in Motivational Interviewing: ELICIT-PROVIDE-ELICIT. There will be an active information exchange with the patients, rather than the transmission of information from a health professional to passive patients as is traditionally the case.

ELICIT: engage the group of patients, usually by asking an open question, to find out what they already know about the topic, or how they feel about it, or perceived information needs and issues of concern or interest. You may also ask permission from the group to provide further information about the given topic. This enables the group to shape the conversation and ensures that it starts from their perspective, rather than that of the health professional. It helps the patients engage with the information in a meaningful way and to apply it to their lives.

PROVIDE: share relevant information with the group in a neutral manner, tailored to what you have learnt about the group from the prior discussion. The health promoter tries to avoid unsolicited and prescriptive advice based on what patients ‘must do’. This information giving component is not an extended lecture, but rather the provision of ‘chunks of information’ within a context of constant dialogue. The health promoter checks that the group has understood the information she has provided, can apply it to their own lives or circumstances and engages with it in a meaningful way. Various group activities or strategies to make the sharing of information more participatory will also be suggested. Supportive educational material, such as pictures or cards, have been developed for selected activities.

ELICIT: ask the group how they might apply this information to their own lives. The purpose is to guide the health promoter on what further information is needed and help the group personalize the information.

In CLOSURE, the health promoter will aim to ELICIT ‘change talk’ from every individual in the group. They will be requested to reflect on and share what they feel they could do to improve the management of an aspect of their diabetes. This goal setting exercise will differ according to each individual and can be at different levels, with some people perhaps feeling that they can only make minor changes and others perhaps setting themselves more challenging goals. Encourage people to set SMART goals, which even if small, are Specific, Measurable (or observable), Achievable, Realistic and within a clear Timeframe.

During this exercise, the health promoter will be required to take notes of the goals set by each individual. She can then use this information to effectively follow up on progress at the beginning of the next session.

The essential communication skills needed for health promoters to facilitate a discussion in this way would be:

- Asking open question
- Summarising
- Elicit-provide-elicit

# SESSION ONE: Understanding Diabetes

## Aim

This session will cover the following topics:

- What diabetes is
- Some of the common myths and facts about diabetes

## Introduction (30 mins)

Time will be needed for people to introduce themselves around the group and respond to an opening question such as:

- What has your experience with diabetes been so far?

## “True or false” game (30 mins)

ELICIT: Possible opening questions for this exercise:

- Have you heard anything about diabetes that you are unsure of?

Any suitable suggestions can be added to the exercise below

PROVIDE:

“Having good information makes managing diabetes easier. Let us take a look at some of the common ideas or beliefs about diabetes. I would now like to hand out some cards. On each card these is a statement about diabetes that may be true or false. You will each receive one and I would then like you to turn to the person next to you and discuss whether you think what is written on your card is true or false.”

After a few minutes ask each person to place their card under the label “true” or “false”. For example you could have a sheet of newsprint with the heading “true” and one with “false” and ask them to put their cards where they think it should be and to explain to the group why they think it belongs there. This should feel fun for the group and in the spirit of a game. It should not feel like a test. Encourage discussion in the group on why the statements are true or false and provide information where needed to make an informed choice. Correct any mis-information.

ELICIT:

At the end a final question can be asked:

- Do you have any further questions about any of the information we have discussed so far?
- Did any of the answers to these statements surprise you or make you think differently about your diabetes?

Further possible questions to extend the group discussion (if there is time):

- Where do most people get their information about diabetes from?
- What can you do to make sure you have accurate information?

## What is diabetes? (30 mins)

ELICIT: Possible opening question to elicit prior knowledge, beliefs and information needs:

- How would you describe diabetes in your own words?
- What questions do you have about how diabetes works?

PROVIDE: Provide information on “what is diabetes” and link to the ideas that have come out of the group

| *For this section, you can use the flipcharts which graphically illustrate the biological processes by which diabetes occurs.*  *The graphics show the human body and the main organs involved in diabetes:*   - *Stomach and intestines, where you swallow and digest food and glucose is absorbed (glucose represented by teaspoons of sugar). Glucose is the fuel your body needs for energy. Excess food and glucose are generally stored as fat.* - *Liver which also makes glucose, especially when glucose is in short supply for example during hard exercise* - *Pancreas an organ in your body that produces insulin (represented by keys).* - *Insulin a hormone that carefully controls the amount of glucose in your blood. It helps your body’s cells take in glucose from your blood stream to use it for energy. Too much fat, especially around the waist, can make cells resistant to insulin.* - *Blood stream which takes glucose and insulin to the rest of the body*   *The graphics show how insulin works on the cells in a normal person, a person with type 2 diabetes and a person with long standing type 2 diabetes*   - *Normal person: Insulin (keys) open the doors to the cells and allow the glucose to go inside and supply energy. Cells are the basic units of life in your body. They require energy in the form of glucose. They make tissues such as muscle. Only a small amount of glucose is left in the blood and only a small amount of insulin is needed.* - *Type 2 diabetes: The insulin (keys) are not always working (insulin resistance) and many of the doors to the cells remain closed. Glucose builds up in the blood and the pancreas makes more and more insulin to try and overcome the resistance.* - *Long standing type 2 diabetes: None of the cells are open and the pancreas no longer produces lots of insulin. The patient is very uncontrolled with lots of glucose in the blood. They probably need insulin to be prescribed in order to get control.* |
| --- |

**Figure 1: Picture of a normal person**


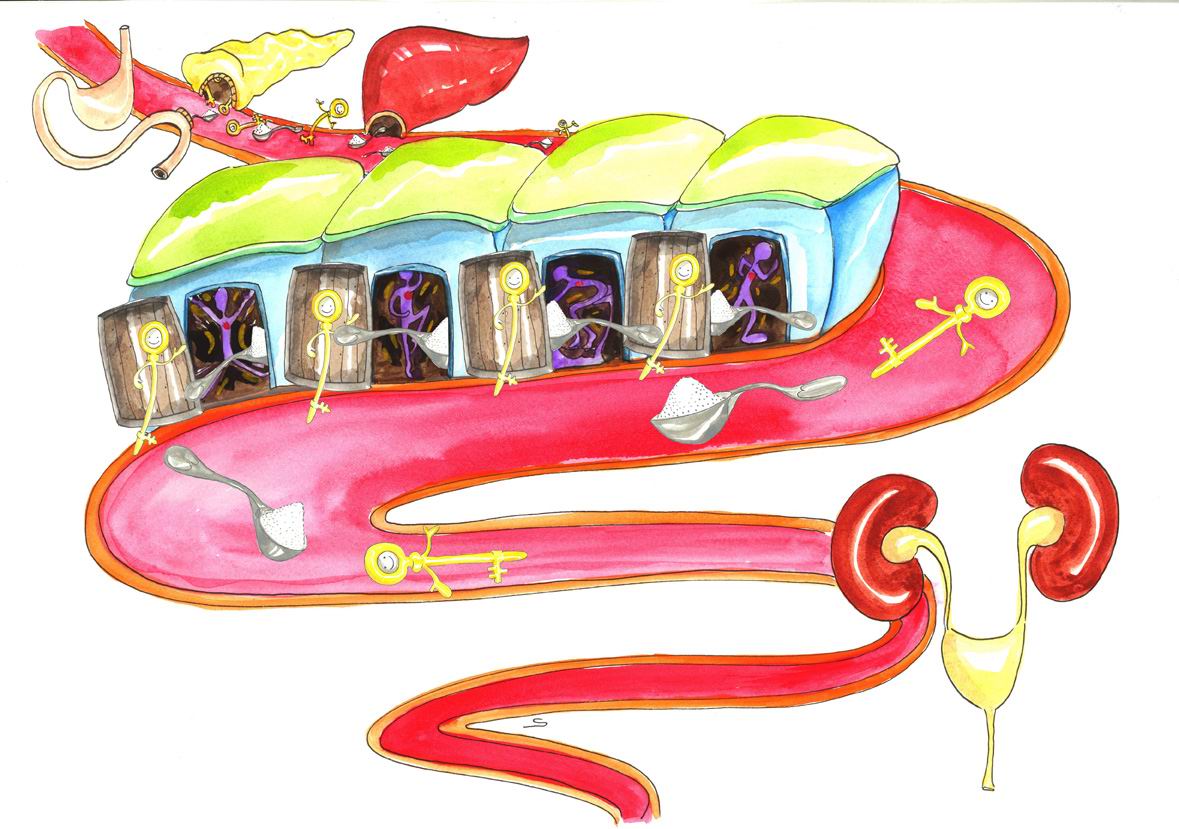


**Figure 2: Picture of someone with type 2 diabetes**


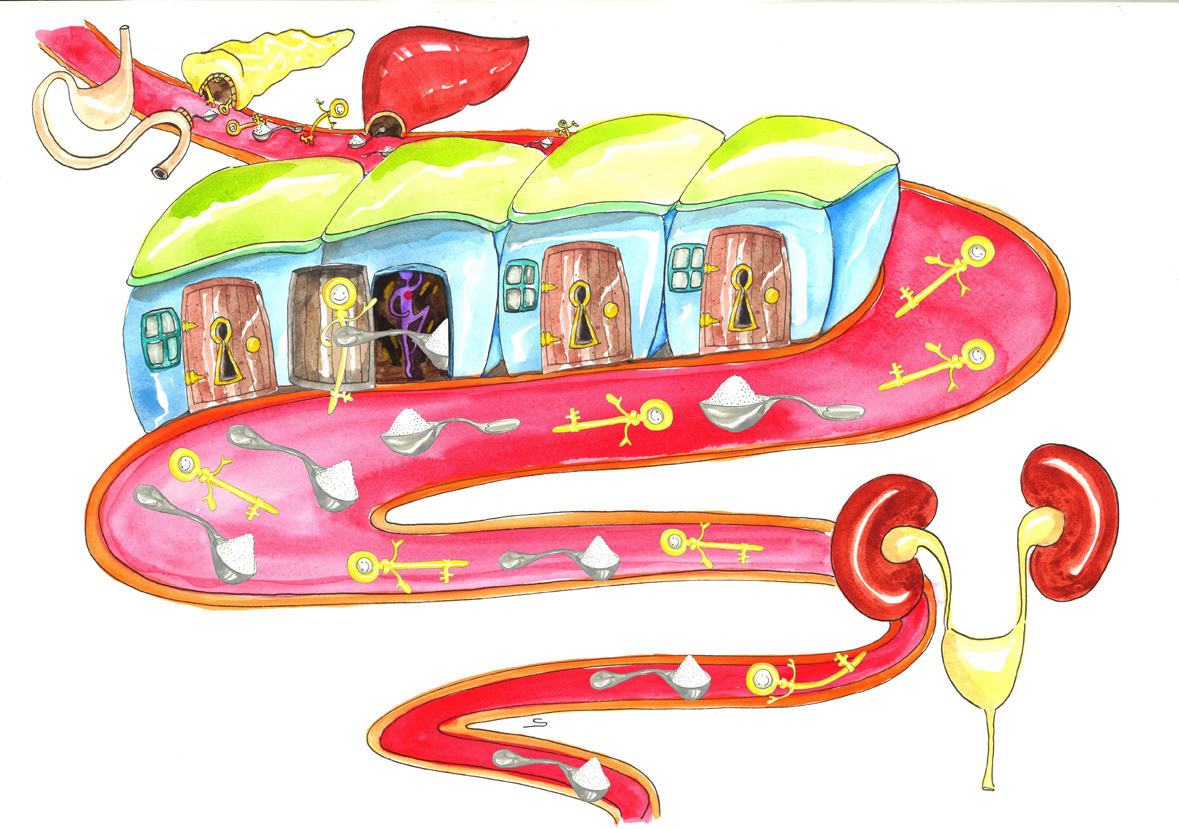


ELICIT: Encourage questions of clarification from the group and ask:

- Before we continue on to the next topic, do you have any questions about what diabetes is or any of the information we have discussed so far?

## Closure: Goal setting (30 mins)

Ask each individual to think of making some change, based on what they have learnt in this session, which would improve their diabetes (it can be a small change….). Ask each one of them to explain how they will put this into action and to share their SMART goal with the group. Inform them that you will ask them about progress next time you see them.

Facilitator can take notes so that she can effectively follow up in the next session.

**Make sure the patients know when to come for the next session**

# SESSION TWO: Living a Healthy Lifestyle

## Aim

This session will cover the following topics:

- What is healthy food
- What are sensible or healthy portion sizes
- How to prepare food
- Timing of meals and snacks
- Physical activity
- Other lifestyle issues (smoking, stress, alcohol)

## Introduction (15 mins)

Ask the group to share their experiences of any changes they tried to make to improve the management of their diabetes since the last session. Ask them what enabled them to make the changes, what they learnt and how they implemented these changes.

## Eating a healthy diet (45-60 mins)

### Food groups (15 mins)

ELICIT:

Facilitator engages group in a discussion by asking them what kinds of foods they usually eat. The facilitator hands out cards with pictures of different types of food and asks the group members to select the ones that they commonly eat (in most weeks). This provides the group with a selection of foods that they are familiar with.

PROVIDE:

| The facilitator then provides information on the 5 food groups (fruits and vegetables; starches and sugars; dairy; protein; fats) using the flipchart provided. |
| --- |

ELICIT:

The group can organize the foods commonly eaten into the 5 food groups.

Does anyone know which of these food groups have the greatest impact on blood glucose?

Does anyone know which of these food groups have the greatest impact on weight / waist circumference?

PROVIDE:

| The facilitator can provide further information to correct or add to the group’s knowledge. |
| --- |

ELICIT:

What are some of the key things that people have learnt regarding their own health eating plans?

### Portion sizes (15 mins)

In addition to what you eat, how much and when you eat are also important when it comes to managing your diabetes. Many people make healthy food choices but still eat too much. How do you know how much to eat?

PROVIDE:

| *The facilitator can provide further information and use one or both of the following tools:*   - *The picture of the plate divided into sections equal to portion size for starches, protein, fat, and vegetables* - *The pictures of the Zimbabwe hand jive – portion of starch is the size of your fist, portion of fruit is the size of your fist, portion of protein is the palm of your hand,*    - *Carbohydrates (starch): choose an amount equivalent to the size of one fist*   - *Fruit use one fist.*   - *Protein: choose an amount equivalent to the size of the palm of your hand and the thickness of your little finger*   - *Vegetables: choose as much as you can hold in both hands. These should be low carbohydrate vegetables – e.g. green or yellow beans, cabbage or lettuce.*   - *Fat: limit fat to an amount the size of the tip of your thumb. Drink no more than 250 ml of low-fat milk with a meal* |
| --- |

ELICIT:

Did anything strike people in the group as particularly relevant to them?

### Timing of meals (5mins)

Now let’s talk about the timing of meals and when you eat.

- How can the timing of your meals affect your blood glucose levels?

PROVIDE:

| Provide information as needed to help answer the above questions. Cover areas such as how many times to eat a day, snacks, what happens if you skip a meal. |
| --- |

### Cooking methods (10 mins)

ELICIT:

Another issue that is important is how you cook your food.

- What cooking methods do people commonly use?
- How does the cooking method impact on your blood glucose or weight/waist circumference?

PROVIDE:

| Provide information as needed to help answer the above questions. |
| --- |

ELICIT:

Before we move onto the next section what are some of the most important things that have struck you personally from this discussion on health eating?

### Meal planning (optional, omit this step if time is limited)

ELICIT:

Divide the participants into 3-4 groups and ask them to use the cards to put together one healthy meal– breakfast, lunch or supper. Put to one side the kinds of foods they should eat only very occasionally or should avoid altogether.

Then the health promoter facilitates a group discussion on the meal plans.

PROVIDE:

| Additional information may be shared in this discussion if appropriate and not already covered.   - Role of salt in hypertension - Effect of alcohol on diabetes - Artificial sweeteners - Food labels - Heart Foundation logo |
| --- |

ELICIT:

Before we move onto the next section what are some of the most important things that have struck you personally from this discussion on health eating?

The following issues may already have come up and can be important to discuss if not yet covered:

- Can people from poorer backgrounds afford to eat healthy food?
- How can one involve the whole family in changing to more healthy food?

## Increasing physical activity (30 mins)

ELICIT:

In addition to healthy eating, keeping active is especially important if you have diabetes. It will help control your blood glucose, reduce your risk for complications, and give you more energy.

- What types of things are you doing when it comes to keeping active?
- How much time each week do you spend being active?
- Approximately how much activity should you get during the week?

PROVIDE:

| *Allow people space to estimate how much time they currently spend on being active*  *Provide information if needed on how much activity is recommended. It is recommended that people with diabetes get at least 150 minutes of aerobic exercise each week (moderate intensity). It is also recommended that people with diabetes do resistance activities 3 times a week or more. Resistance activities are activities that use muscular strength to move a weight or work against a resistant load.*  *It may also be possible to demonstrate a simple activity or exercise in the group* |
| --- |

ELICIT:

Keeping active will help you feel better and help you manage your diabetes. It is good to find a routine that you can follow consistently. Think of what you do on a daily basis.

- How could you increase activity in your daily routine?
- What challenges do you face in keeping active?
- How might you overcome these challenges to keeping active?

## Other lifestyle issues (15 mins)

ELICIT:

A discussion of these topics can be prompted by asking the group how they think alcohol, smoking and stress are related to the control of diabetes.

PROVIDE:

| - *Alcohol; what is moderate use for men and women (Low risk drinking in men is up to 3 standard drinks a day and below 15 standard drinks a week, women up to 2 standard drinks a day and below 10 standard drinks a week); drinks which have lots of carbohydrates (traditional beer); how alcohol affects the ability of the liver to release glucose when needed (refer back to picture from ‘what is diabetes’); how getting drunk affects your ability to manage your medication and diet properly.* - *Stress: the role of stress in increasing blood sugar and blood pressure.* - *Smoking: how smoking causes damage to the vascular system, which adds to one’s already increased risk of cardiovascular disease (heart attack and stroke)* |
| --- |

ELICIT:

Are there any other questions about these issues?

Are there people in the group who would like more help with any of these issues?

PROVIDE:

| *Health promoter needs to be able to direct people who would like further help with these issues to other resources or make arrangements for them to see her at another time. Educational and self help materials on smoking and alcohol are available for those who need them.* |
| --- |

## Closure: Goal setting (15mins)

Ask each individual to think of making some change, based on what they have learnt in this session, which would improve their diabetes (it can be a small change….). Ask each one of them to explain how they will put this into action and to share their SMART goal with the group. Inform them that you will ask them about progress next time you see them.

Facilitator can take notes so that she can effectively follow up in the next session.

**Make sure the patients know when to come for the next session**

# SESSION THREE: Understanding your medication

## Aim

This session will cover the following topics:

- Using medication to control diabetes
- Considerations when using medication
- Dealing with hypoglycaemia (low blood glucose)

## Introduction (30 mins)

Ask the group to share their experiences of any changes they tried to make to improve the management of their diabetes since the last session. Ask them what enabled them to make the changes, what they learnt and how they implemented these changes.

## Managing diabetes with medication (45 mins)

ELICIT:

Possible questions:

- Most of you will be taking medication for diabetes, what has your experience been like?
- What questions do you have about the medication that you are taking for diabetes?

Write up any issues or questions to be addressed on newsprint.

PROVIDE:

| *You as the facilitator need to have examples of the medication used by the patients with you in this session. Show each medication one at a time and explain what each medication does, how it works and how it should be taken. It may help to remind people of the pictures used in ‘what is diabetes’ and show how the medications help reduce blood glucose.*  *Biguanides (Metformin): Increase the sensitivity of cells to insulin*  *Sulphonylureas (Glibenclamide, Glicazide): Enable the pancreas to make more insulin*  *Injectable Insulin: Increases the amount of insulin in the body and lowers blood glucose*  *Address all their issues/questions one by one. Be sure to address questions relating to how to deal with special situations such as going away on holiday and fasting if these do not come up spontaneously.*  *Health promoter should be prepared to comment also on antihypertensive and lipid medication*  *Give information in small Chunks and Check for understanding.*  *It may also be important to discuss how patients with other conditions such as HIV or TB should manage taking multiple types of medication.* |
| --- |

ELICIT:

- Will any of this help you with taking your own medication?
- Do you have any further questions about any of the information we have discussed so far?

Additional questions that can be asked to extend this section:

- Do you have any questions about how to get your medication at this clinic?

## Dealing with low blood glucose (15 mins)

ELICIT:

As we have discussed, often medication treatment helps to lower your blood glucose levels. Sometimes, in doing this, your blood glucose gets too low. So, how do you know if your blood glucose is too low? Has anyone experienced what is it like to have a low blood glucose?

PROVIDE:

| *Correct the group’s ideas or add to them. Typical signs and symptoms of low blood glucose are hunger, headaches, looking pale or sickly, trembling or shaking, unable to concentrate.*  *Check for the groups understanding. Use the newsprint to summarise.* |
| --- |

ELICIT:

What should you do if your blood glucose gets too low?

What foods can you eat to bring your blood glucose back up?

PROVIDE:

| *Correct the group’s ideas or add to them. Your blood glucose levels should stay above 4.0mmol/L, however most people will not have a monitor to check this at home. Evidence suggests that 15g of glucose will most likely return blood glucose to normal levels and relieve symptoms of low blood glucose in most people. Make sure the participants identify sources of 15g of glucose readily available to them. Discuss the most appropriate choices with the group (e.g. fruit juice would be better than chocolate due to the fat content in chocolate). Severe hypoglycaemia should be treated by 20g of glucose. If you suspect low blood glucose it is best to treat yourself, even if you are not sure.* |
| --- |

ELICIT:

Having discussed all of this, what might you do differently when you have a low blood glucose?

Do you have any remaining questions about how to recognize and treat low blood glucose?

## Symptoms of high blood glucose (15 mins)

ELICIT:

Let us start by talking about what it feels like to have high blood glucose.

- What does it feel like when your blood glucose is high?

PROVIDE:

| *Provide information about other symptoms and clarify misconceptions. Clarify the different symptoms for high vs. low blood glucose. Low blood glucose was covered in a previous session.* |
| --- |

ELICIT:

When you come to the clinic you usually have a finger prick blood test to measure your blood glucose.

- What sort of numbers do you usually get in the clinic for your blood glucose?
- What do you think your blood glucose should be?

PROVIDE:

| *Fasting (or before meals) blood glucose levels should be between 4.0 and 7.0 mmol/L. Two hour postprandial (or after meals) blood glucose levels should be between 5.0 and 10.0 mmol/L.* |
| --- |

ELICIT:

Now that you know these are the normal levels for blood glucose how do you feel about your own blood glucose levels?

What are some of the things that cause high blood glucose?

PROVIDE:

| *This is an opportunity for people to remember and express some of the things already covered and for the health promoter to reinforce them.* |
| --- |

ELICIT:

What do you think you can do to try and avoid high blood glucose levels?

PROVIDE:

| *This is an opportunity for people to remember and express some of the things already covered and for the health promoter to reinforce them.* |
| --- |

## Closure: Goal setting (30 mins)

Ask each individual to think of making some change, based on what they have learnt in this session, which would improve their diabetes (it can be a small change….). Ask each one of them to explain how they will put this into action and to share their SMART goal with the group. Inform them that you will ask them about progress next time you see them.

Facilitator can take notes so that she can effectively follow up in the next session.

**Make sure the patients know when to come for the next session**

# SESSION FOUR: Preventing complications

## Aim

This session will cover the following topics:

- The dangers of high blood glucose
- Recognising and controlling high blood glucose
- What medical assessments need to be done to effectively identify and prevent complications and when these should be done

## Introduction (15-30 mins)

Ask the group to share their experiences of any changes they tried to make to improve the management of their diabetes since the last session. Ask them what enabled them to make the changes, what they learnt and how they implemented these changes.

## Complications (15 mins)

ELICIT:

Having diabetes and high blood glucose puts you at risk for many complications in all different parts of the body.

- What are some of the complications that you know about or may even have experienced yourself?

PROVIDE:

| *Ensure that all the key complications are listed and be able to explain them:*  *Vascular disease: People with diabetes are at increased risk of vascular disease that can impact the heart, brain and blood vessels supplying the legs and feet. This increases the risk of heart attack, stroke, dementia, impotence, and other complications related to poor circulation. People with diabetes often have high blood pressure and abnormal lipids (cholesterol) which also increase the risk of vascular disease.*  *Kidney disease: Too much glucose in the blood can damage the small blood vessels in the kidneys. When the blood vessels in the kidneys are damaged, the kidneys cannot work properly and could leak albumin (protein) into the urine. This may be an early sign of kidney failure.*  *Eye complications: People with diabetes are at risk of developing a complication called retinopathy that affects the blood vessels in the seeing part of the eye. This may lead to visual impairment and blindness. People with diabetes are also at increased risk of cataracts and glaucoma.*  *Diabetic neuropathy and nerve damage: Neuropathy means damage to nerves that run throughout the body, including nerves to or of the feet, heart, and other organs. This type of nerve damage can cause loss of feeling in the feet and impotence.*  *Foot complications: People with diabetes can develop many different foot problems. Foot problems most often happen when there is nerve damage in the feet or when blood flow is poor. Smoking can increase the risk of amputation.*  *Depression: Feeling down once in a while is normal. But some people feel a sadness that just won’t go away. Life seems hopeless. Feeling this way most of the day for two weeks or more is a sign of serious depression*  *Explain how the goals of treatment are to consistently control blood glucose levels so that you feel better and avoid or delay the complications of diabetes in the long term. Focusing on healthy eating, being active and medication options can help someone get back in control and live their best life.* |
| --- |

## Foot care (10 mins)

ELICIT:

Damage to the feet is a common problem with diabetes. As we have discussed some people develop infections, ulcers or even need amputations. There are things you can do to prevent problems with your feet. What are these?

PROVIDE: Handout the list of do’s and don’ts for foot care and allow people to read and ask questions

ELICIT:

Having read through the list, do any of these things look practical or useful for you?

## Understanding the clinic and test results (30 mins)

ELICIT:

When you come to the clinic the doctors and nurses are meant to look for any signs of these complications. Sometimes the complications can be detected early and treated before they become too serious. Some tests are done at every visit and some only once a year.

- What complications have you been screened for – what tests have you done?

PROVIDE:

| List the group’s suggestions and add any that are not mentioned.   - Blood pressure test for hypertension (vascular disease) - Urine test (for protein) for kidney disease - Eye test (vision and retina) for eye disease - HbA1c blood test for overall control of blood glucose - Foot examination of skin, nerves and pulses (nerve damage and vascular disease) - Weight and Body Mass Index and/or waist circumference (for overweight/obesity) - Total cholesterol for abnormal lipids/fats (vascular disease) - ECG (Electrocardiogram tracing of the heart muscle) for heart disease |
| --- |

ELICIT:

- How can you ensure that you are getting screened and that any complications you might have are detected early?

PROVIDE

| *The health promoter may need to provide information on how things are organized at this clinic*  *Ask the group what happens when they attend the clinic for their 3 and 6 months assessments. Explain what screening tests are supposed to happen and how often. Encourage patients to ask the health care providers to explain their results and assist them is setting feasible targets.*  *At this point, the health promoter will hand out a 1-page summary, prepared by the research team, with all the patient’s results from the screening tests performed in this study (HbA1c, total cholesterol, waist circumference, blood pressure) for each study participant. This will list the ideal levels, the actual levels and then have a column for my goals or target levels.*  *Explain what screening tests were done by the research team and ask them to look at the readings they have on their own handout. Ask them what they know about these readings and what constitutes an ideal reading. Emphasise that the Hb1Ac test is the best indicator of blood glucose levels and should be done at least once a year. Explain the last column is for them to set their own targets for the next year.*  *Emphasise that any movement towards the ideal levels represents a significant decrease in risk of complications (even small improvements can have large benefits). Each person can have their own unique targets for the year.* |
| --- |

ELICIT:

Does this make sense to you?

What questions do you have about setting realistic targets in the last column?

Discuss your results with your neighbor and what targets you think would be realistic for you over the next year. Write your targets on the page.

Ask each individual to think of making some change, based on what they have learnt in this session, which would improve their diabetes (it can be a small change….). Ask each one of them to explain how they will put this into action and to share their goal with the group.

## Closure of the course (30 mins)

This activity will constitute closure for all four sessions.

Go around the group and ask people to respond to the following two questions:

- When you look back at the 4 education sessions what changes have you made to the way you look after yourself and your diabetes?
- What has it been like participating in this group education?

Certain logistical study issues will be raised at this time. Explain that further study assessments will be undertaken at their next visit in 1 months time and again at their clinic visit in 6 months time.

## Graduation (30 mins – this is in addition to the usual 2 hrs)

Participants will all be awarded with a certificate to acknowledge their participation.

# Guidelines for guiding in groups^[[1]](#footnote-1)^

## Avoid traps

- Don’t conduct multiple individual consultations in a group setting
- Avoid question and answer sessions conducted by you, the expert
- Avoid allowing the group to become either too unfocused or too serious

## Golden rules

- Remember your goal; to bring everyone together to focus on a topic and gain support from one another
- Link individual stories to the topic and experience of others. Extract the essence of the patient’s story and broaden it out.
- Encourage the quiet, soften the loud. For example you might ask quiet members to summarise some part of the group discussion and discuss how it fits for them. You might ask talkative members to summarise their points and pose a question to the group.
- Minimize negative interactions. Participants can overdo giving advice to others or even confront their apparent “denial” or excuse for avoiding change. Don’t let negative interactions escalate. Remain empathic and supportive, but act immediately to regain control when group processes are going in a negative direction. Ask participants to reframe their advice to others into statements of “what has worked for me”
- Keep the focus of the group on enhancing motivation to change, increasing hope, and reducing the sense of burden that change imposes. Don’t allow the groups to become complaint sessions.

## Useful strategies

- Past successes: Focusing on things that participants have achieved can help to restore self-confidence and spark creativity in regard to the current change
- Ambivalence: Focusing on participants mixed feelings about change can help reduce defensiveness while preparing them to both initiate change and prevent relapse
- Values: Supporting participants in examining how their current behavior fits with their core values can enhance motivation to change and help them find an internal source of direction to rely on when the status quo is threatening to take the upper hand
- Looking forward: Helping participants envision a better future, rather than falling into a pattern of begrudgingly acknowledging and exploring past failures, can positively affect the relationship between the participants and their struggles to change
- Exploring strengths: Eliciting participants’ sense of their own strengths can enhance their self esteem and help them find internal resources that can support their current change effort.
- Planning change: using discussion and worksheets to plan change alone, in pairs, or as a large group can help transform vague motivation into concrete plans that help in initiating and maintaining change. Encourage participants to state one small change they are committing to rather than making a grand but vague plan. Follow up on how these commitments went in the next group meeting.
- Exploring importance and confidence: using importance and confidence rulers to examine the relationship between participants and their change plans helps participants to see that these internal cognitive and emotional elements can either support them in their change or hold them back.

*This project is supported by a BRIDGES Grant from the International Diabetes Federation. BRIDGES, an International Diabetes Federation project, is supported by an educational grant from Eli Lilly and Company.*

1. Drs Karen Ingersoll and Chris Wagner, page 171-2, Motivational Interviewing in Health Care, 2008. [↑](#footnote-ref-1)
